# Adaptation and validation of the Wylie cephalometric analysis for three-dimensional assessment of canine occlusion in Labrador retrievers

**DOI:** 10.3389/fvets.2026.1955587

**Published:** 2026-09-16

**Authors:** Katherine E. Kudrna, Tsung-Han Tu, Scott J. Hetzel, Jason W. Soukup

**Affiliations:** 1 Dentistry and Oromaxillofacial Surgery, Department of Surgical Sciences, School of Veterinary Medicine, University of Wisconsin-Madison, Madison, WI, United States; 2 Department of Biostatistics and Medical Informatics, School of Medicine and Public Health, University of Wisconsin-Madison, Madison, WI, United States

**Keywords:** 3D cephalometry, canine malocclusion, cephalometry, craniofacial morphology, veterinary orthodontics, Wylie analysis

## Abstract

**Introduction:**

In veterinary medicine, we lack a quantitative method for assessment and classification of occlusion. Current systems of occlusion classification are subjective, limiting the ability for precise treatment planning.

**Methods:**

A medical record search identified 146 head CTs from Labrador Retrievers from 2007–2022. 3D computer models of skulls were created from DICOM files. Thirty standardized anatomic landmarks were placed on each model to perform cephalometric analysis. A cohort of 96 dogs was used to establish breed-specific reference values, with assessment for sexual dimorphism prior to standardization. The remaining 50 dogs served as a validation cohort for the Wylie cephalometric analysis to quantify maxillomandibular length discrepancy. Facial angles analysis and three ratio-based measurements (MXPM4: MNM1, MXK9: MNK9, and MX: MN) were calculated for all samples.

**Results:**

Craniofacial measurements showed significant sexual dimorphism and showed allometric scaling with weight. There was no significant difference between normalization and validation groups. Wylie score clustered near zero (SD = 3), indicating minimal maxillomandibular discrepancy in dogs with normal occlusion. The mean ANB angle was 7.8° ± 1.5°. Ratio-based measurements assessing maxillary-to-mandibular relationships were as follows: MXPM4: MNM1 ratio was 1.1, the MXK9: MNK9 ratio was 0.9, and the MX: MN ratio was 1.0 across all individuals. The ratio-based measurements correlated strongly with the Wylie score.

**Discussion:**

Human cephalometric analyses can be adapted for use in canine patients. This study established a reference standard for cephalometric analysis in Labrador Retrievers as a representative of mesaticephalic canine skulls. The Wylie analysis and ratio-based measurements represent a quantifiable method of occlusion assessment.

## 1 Introduction

Malocclusions are common in dogs and may be broadly classified according to whether the discrepancy arises primarily from abnormal tooth position (dental malocclusion) or from altered jaw growth and spatial relationships (skeletal malocclusion) (1). Skeletal malocclusions may result in periodontal disease, abnormal tooth wear, endodontic disease, impaired oral function, temporomandibular joint (TMJ) disease, and chronic pain (2, 3). In dogs, Class 2 malocclusion, characterized by relative mandibular retrusion, frequently leads to impingement of the mandibular canine teeth on the hard palate, causing mucosal trauma, pain, and in severe cases, oronasal communication (4). Altered occlusal forces associated with skeletal discrepancies may also contribute to degenerative TMJ disease (5–7). In severe cases, treatment with orthodontia alone is insufficient, and conventional salvage procedures such as dental extraction or crown reduction may relieve pain but fail to correct the underlying skeletal imbalance (4). Consequently, definitive understanding of abnormal jaw relationships and their correction remain an area of needed development in veterinary medicine.

In human orthodontics, malocclusions have traditionally been categorized using the Angle classification system, which defines Class I, II, and III malocclusions based on the anteroposterior relationship of the maxillary and mandibular first molars (8) (Figure 1). While this system provides a simple descriptive framework, it does not necessarily directly characterize the underlying skeletal relationships and patients may exhibit normal molar relationships despite maxillomandibular skeletal discrepancies. These limitations led to the development of cephalometry, a quantitative method for assessing craniofacial skeletal relationships using standardized anatomic landmarks and reproducible linear and angular measurements (8, 9) (Figure 2).

**Figure 1:**
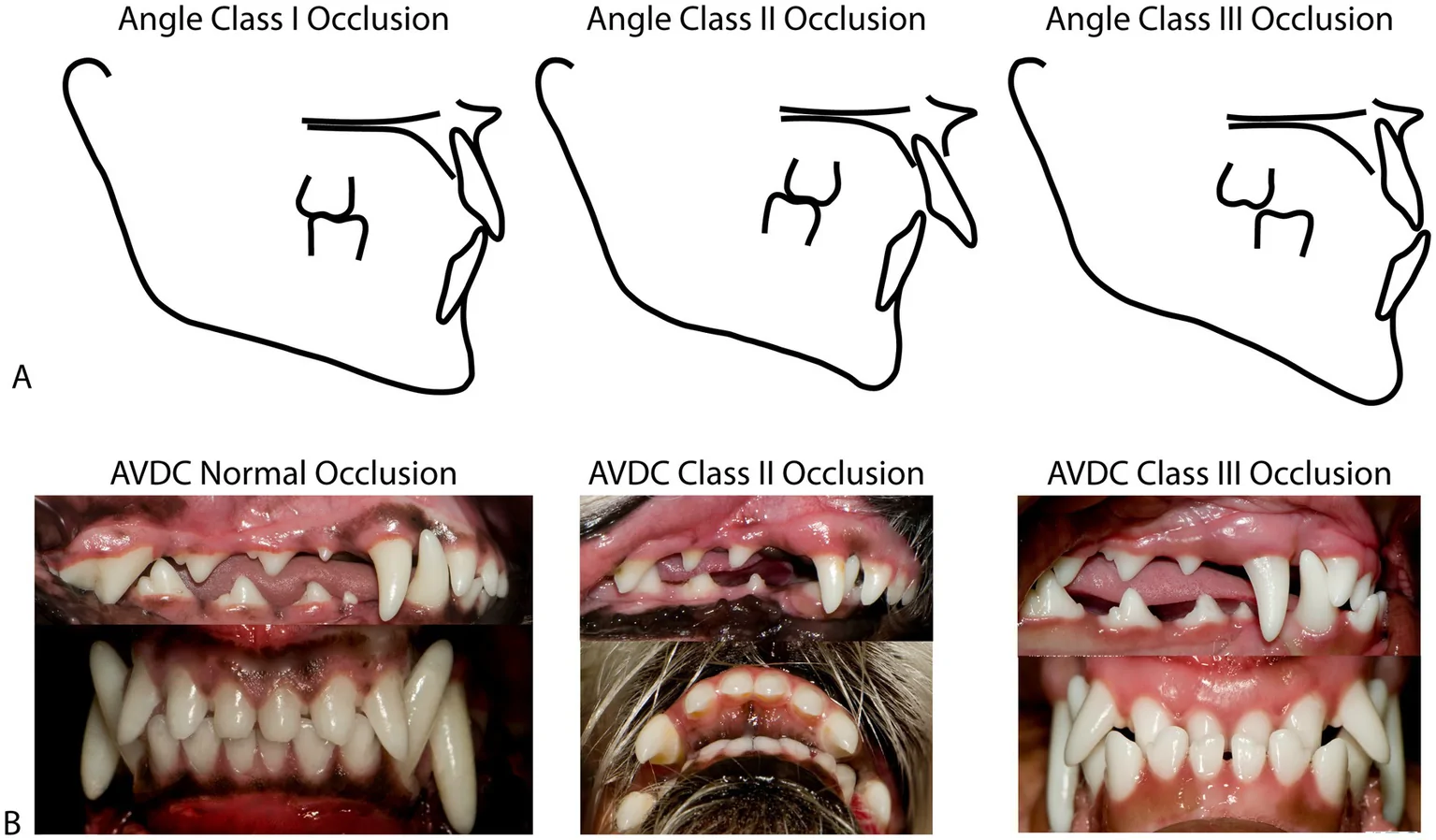
Comparison between the human Angle **(A)** and veterinary AVDC **(B)** classification system for malocclusions (1).

**Figure 2:**
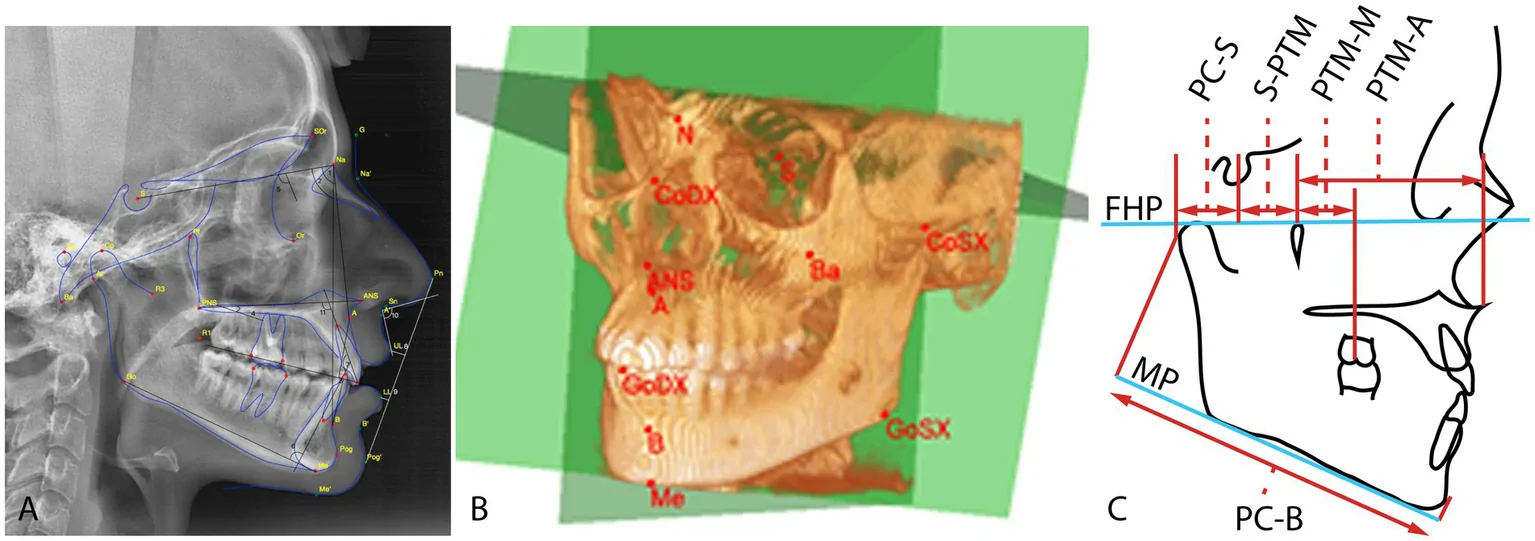
Examples of traditional 2-D **(A)** (37) and 3-D **(B)** cephalometry (16). The Wylie cephalometric analysis system is represented in **(C)**.

Numerous cephalometric analysis systems have been introduced (e.g., Downs, Steiner, McNamara, and Wylie), each emphasizing different aspects of craniofacial morphology (8–12). Most integrate evaluation of anteroposterior, vertical, and angular relationships among the cranial base, maxilla, mandible, and dentition (8). The McNamara analysis, for example, focuses on maxillomandibular position relative to the cranial base (12), whereas the Downs analysis incorporates angular relationships to describe facial pattern and growth direction (11, 13). The Wylie analysis is distinctive in emphasizing linear horizontal proportional relationships among the maxilla, mandible, and cranial base, expressing skeletal discrepancies as deviations from a neutral reference value derived from individuals with normal occlusion (9) (Figure 2). Rather than relying on a single composite score, the analysis localizes the anatomic contributors to malocclusion, enabling precise characterization of skeletal and dental abnormalities. The proportional emphasis of cephalometric frameworks in the Wylie analysis further supports assessment of overall craniofacial harmony rather than isolated positional discrepancies alone.

In veterinary dentistry, malocclusions are classified using the American Veterinary Dental College (AVDC) system, which categorizes occlusal relationships into Class 1 (neutroclusion), Class 2 (mandibular distoclusion), Class 3 (mandibular mesioclusion), and Class 4 (maxillomandibular asymmetry) (1) (Figure 1). Unlike the human Angle classification system, the AVDC classification emphasizes incisor and canine relationships and de-emphasizes the maxillomandibular carnassial teeth relationship (1). Importantly, the AVDC framework does not define quantitative thresholds distinguishing normal occlusion from Class 2 or Class 3 malocclusion, nor does it provide a standardized description of maxillomandibular carnassial tooth relationships analogous to the molar reference used in humans (1). As a result, classification of borderline and complex cases remains subjective, limiting diagnostic consistency, research comparability, and treatment planning. Additionally, existing veterinary classification systems provide limited insight into the structural contributors to malocclusion and offer little guidance for orthodontic intervention.

Historically, human cephalometric analysis was performed using two-dimensional lateral skull radiographs which are subject to projection error, superimposition of anatomical structures, and variability in head positioning (8). Advances in computed tomography (CT), three-dimensional reconstruction, and computer-assisted modeling allow cephalometric analysis to be performed in three dimensions using precisely defined anatomic landmarks (14–18) (Figure 2). These techniques eliminate magnification error, permit direct measurement of spatial relationships, and enable reproducible skull orientation using standardized reference planes. In human cephalometry, the Frankfort horizontal plane (FHP) has been widely adopted as a biologically relevant reference for head positioning (19). In dogs, qualitative evaluation of natural head posture in wolves, the evolutionary reference species for domestic dogs, suggests that FHP closely approximates natural cephalic orientation of canids in the wild, supporting its use for canine cephalometric measurement (20) (Figure 3).

**Figure 3:**
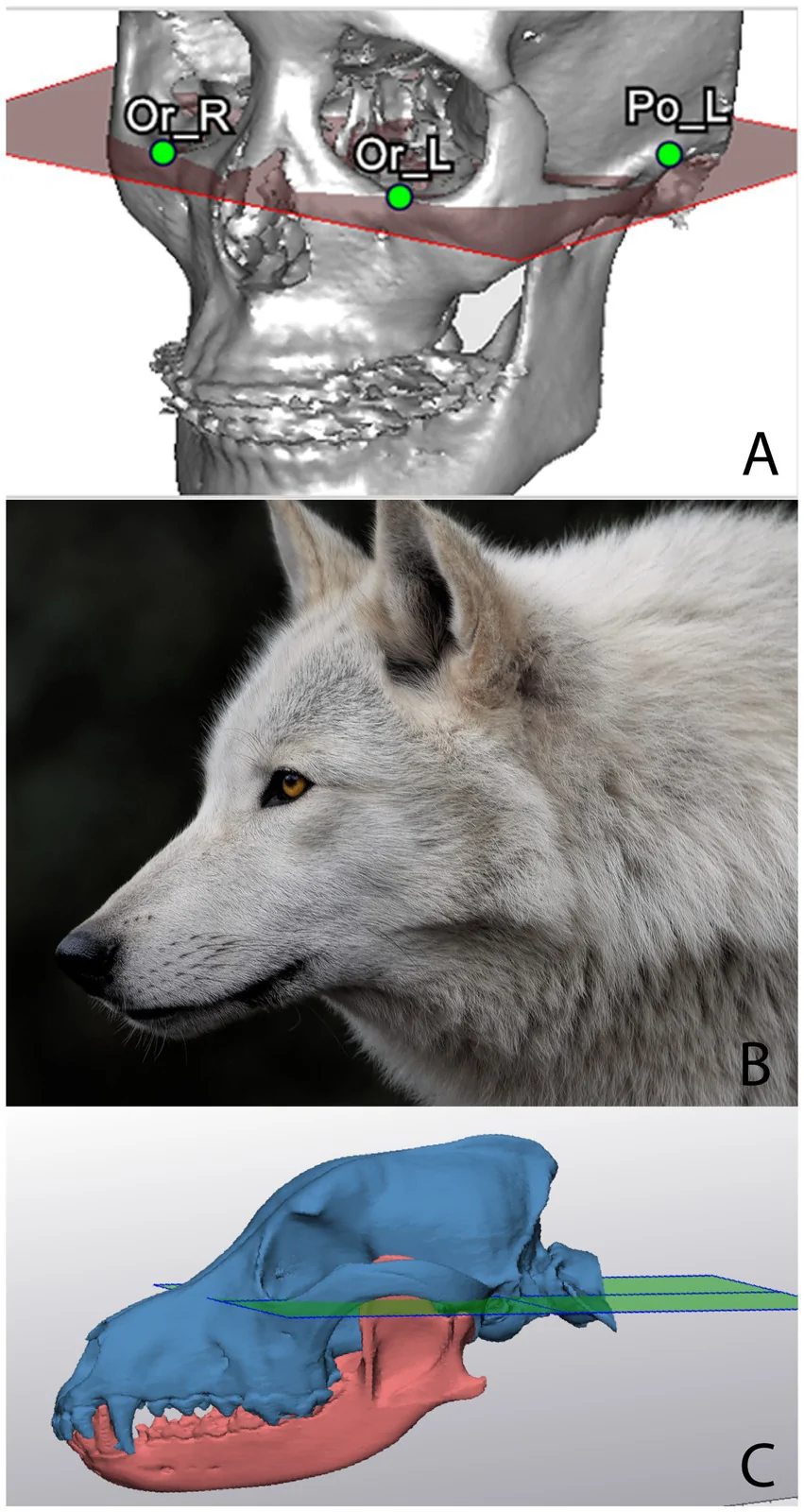
Representations of the Frankfort horizontal plane (FHP) in a human 3-D model defined by the left and right Orbitale (Or_L and Or_R) and the left porion (Po_L) (15) **(A)** and the analogous FHP in a dog 3-D model **(C)**. Note that the FHP is a good representation of normal head position as depicted in a wolf (by ambquinn via Pixabay License) **(B)**.

Importantly, in both humans and dogs, dental and skeletal malocclusions do not always coexist; individuals may exhibit normal dental occlusion despite underlying skeletal discrepancies (21). This dissociation underscores the limitations of purely dental classification systems and highlights the need for objective skeletal assessment in veterinary patients.

These considerations reveal a fundamental gap in veterinary dentistry: the absence of a quantitative, reproducible framework for defining normal and abnormal maxillomandibular relationships in dogs. Adapting validated human cephalometric principles to three-dimensional CT imaging offers a practical approach to establishing canine-specific skeletal standards. Among existing analytic systems, the Wylie proportional framework, by emphasizing linear skeletal relationships and minimizing reliance on vertical facial dimension, may be particularly suited to canine craniofacial morphology.

Accordingly, the objective of this study was to adapt the Wylie cephalometric analysis for use in dogs using CT-based three-dimensional modeling. Mesaticephalic dogs with clinically normal skeletal occlusion were used to establish reference standards, which were subsequently applied to an independent cohort to validate the method. We hypothesized that dogs with normal skeletal occlusion would yield a Wylie analysis score consistent with maxillomandibular harmony (i.e., approximating zero). In addition, proportional analyses (ratio-based measurements) were developed to quantify key maxillomandibular and dental relationships, including the maxillary fourth premolar to mandibular first molar relationship, maxillary to mandibular canine relationship, maxillary to mandibular length ratio, and the ANB angle, which were hypothesized to approximate unity in dogs with normal occlusion.

## 2 Materials and methods

### 2.1 Case selection

A retrospective medical record search was performed using the database of the University of Wisconsin–Madison Veterinary Medical Center. Canine patients were included if Labrador Retriever was listed as the breed and a head computed tomography (CT) study had been performed between April 2007 and July 2022. Inclusion criteria consisted of 1. complete head CT encompassing the nasal planum through at least the first cervical vertebra, 2. adequate image resolution to allow placement of predefined anatomic landmarks, 3. medical records including a physical examination with a reported oral exam sufficient to determine a normal occlusal relationship. Exclusion criteria included incomplete CT studies or significant craniofacial pathology that precluded landmark placement on one or both sides of the skull or that caused craniofacial deformation.

### 2.2 Image acquisition, processing, and 3D reconstruction

Computed tomographic studies were acquired and reconstructed using a bone reconstruction kernel/algorithm to optimize edge definition of osseous structures. Digital Imaging and Communications in Medicine (DICOM) datasets of studies were imported into dedicated medical image segmentation and modeling software (Materialise Mimics Core, v. 27.0, Materialise NV; Leuven, Belgium). Skull masks were generated from the bone window using automatic global thresholding (226–3071 HU). The mask was manually split to generate separate masks for the maxilla, mandible, as well as other structures outside of the region of interest (e.g., hyoid apparatus, endotracheal tube, which were subsequently removed prior to analysis). Following segmentation, bone masks of the maxilla and mandible were converted to a 3D surface using Mimics’ Calculate 3D function as previously described (22). Prior to this step, a mask smoothing operation was applied as needed, at the discretion of the operator, to reduce voxel-related stair-step artifact on the mask surface while preserving anatomical accuracy of the underlying cortical bone; masks that did not exhibit visible stair-step artifact were left unsmoothed so as not to alter fine surface detail unnecessarily. The mandible was repositioned into occlusion relative to the maxilla using software-based rotational manipulation centered upon the TMJ to effectively close the mouth. In-silico models were subjectively evaluated for any subjective evidence of skeletal malocclusion according to the AVDC malocclusion classification descriptions.

### 2.3 Landmark identification and orientation

Thirty craniofacial landmarks were assigned to each skull model within Mimics (Table 1; Figure 4). Landmark selection was adapted from established human cephalometric landmarks. Midline landmarks (Basion, Sella, Nasion, Posterior Nasal Spine, Prosthion [A-point], and B-point) were placed once per skull, while bilateral landmarks were placed on both the right and left sides when possible. Dental landmarks were placed at the intersection of the distal aspect of the tooth and the alveolar margin of the maxillary third incisor, canine, fourth premolar teeth as well as the mandibular canine, first molar, and third molar teeth. Some landmarks were placed for use in future studies and were not utilized in this study (e.g., Posterior Nasal Spine). To standardize skull orientation, the FHP was constructed using the right Porion and bilateral Orbitale points. This plane was used to orient the skull in a standardized reference position in order to accurately assign some anatomic landmarks.

**Table 1:** Craniofacial landmarks with abbreviations and definitions used in cephalometric analysis.

Landmark	Abbreviation	Definition
Basion	Ba	Point at which the midsagittal plane intersects the lowest point on the anterior margin of the foramen magnum
Porion	P	Most superior and lateral point on the bony external auditory meatus
Sella	S	Midpoint of the sella turcica (hypophyseal fossa of the sphenoid bone)
Orbitale	Or	Most inferior point on the orbital margin
Posterior Maxillary Tuberosity	PTM	Rounded distal end of the alveolar process of the maxilla, posterior to the last molar
Posterior Nasal Spine*	PNS	Posterior projecting process formed by the united median ends of the palatine bones
Nasion	N	Point at which the midsagittal plane intersects the most anterior point of the nasofrontal suture
Prosthion (A-Point)	A	Most anterior and inferior point of the maxillary alveolar process between the central incisors
Maxillary Third Incisor (Distal Alveolar Point)	MXI3	Point placed at the intersection of the distal aspect of the maxillary third incisor tooth and the alveolar process
Maxillary Canine (Distal Alveolar Point)	MXK9	Point placed at the intersection of the distal aspect of the maxillary canine tooth and the alveolar process
Maxillary Fourth Premolar (Distal Alveolar Point)	MXPM4	Point placed at the intersection of the distal aspect of the maxillary fourth premolar tooth and the alveolar process
Posterior Condyle	PC	Most posterior aspect of the mandibular condylar process, identified with the Frankfort horizontal plane parallel to the horizontal axis
Angular Process	AP	Most caudodorsal point of the angular process of the mandible
Middle Mental Foramen	MMF	Most distal point of the mental foramen
B-point	B	Deepest concavity on the anterior surface of the mandibular symphysis between the central incisors
Mandibular Canine (Distal Alveolar Point)	MNK9	Point placed at the intersection of the distal aspect of the mandibular canine tooth and the alveolar process
Mandibular First Molar (Distal Alveolar Point)	MNM1	Point placed at the intersection of the distal aspect of the mandibular first molar tooth and the alveolar process
Mandibular Third Molar (Distal Alveolar Point)	MNM3	Point placed at the intersection of the distal aspect of the mandibular third molar tooth and the alveolar process, representing the transition from mandibular body to ramus

*Denotes landmarks not utilized for measurements in the current study.

**Figure 4:**
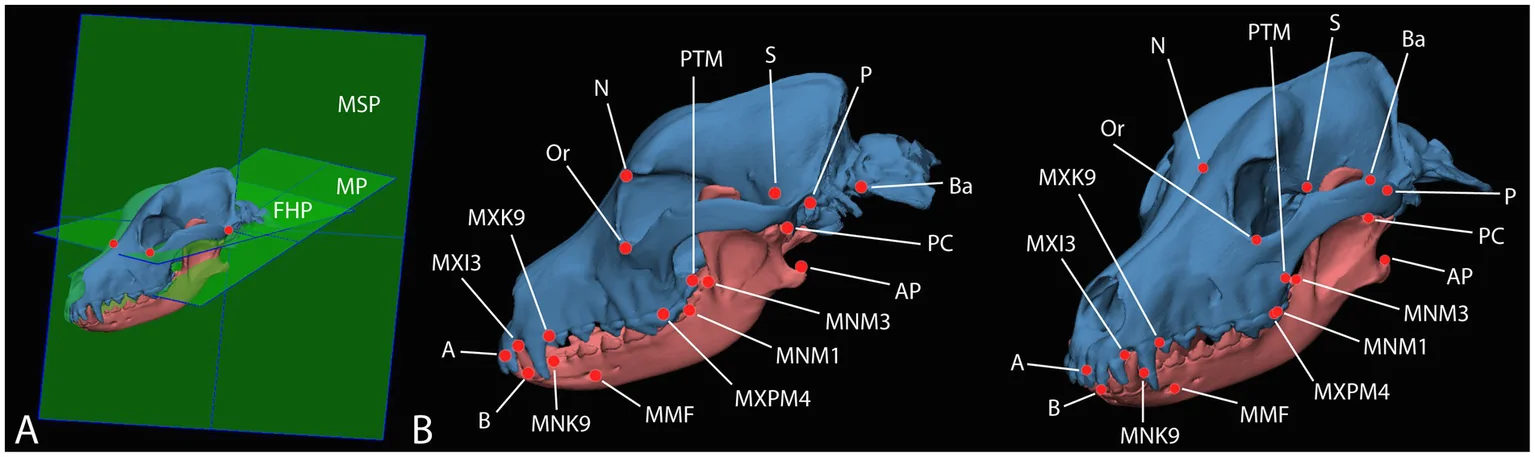
**(A)** Representation of the three planes utilized for orientation in the present study: median sagittal plane (MSP), mandibular plane (MP), and the Frankfort horizontal plane (FHP) **(B)** Anatomical landmarks utilized in the present study. Abbreviations and definitions can be found in Table 1.

### 2.4 Plane construction and measurements

The segmented maxilla, mandible, and associated landmarks were exported to 3-Matic (Materialise 3-Matic v. 19.0, Materialise NV; Leuven, Belgium) for construction of new reference planes and linear measurements. Three planes were created for each skull (Figure 4):

- Midsagittal Plane (MSP): Defined using Basion, Nasion, and Prosthion.
- Frankfort Horizontal Plane (FHP): Defined by the right Porion and bilateral Orbitale points, translated to the MSP origin and rotated to be orthogonal to the MSP.
- Mandibular Plane (MP): Defined using the bilateral angular processes and right middle mental foramen, translated to the MSP origin and rotated to be orthogonal to the MSP.

Thirteen unique linear measurements between select craniofacial landmarks were obtained. (Table 2). Right-sided landmarks were used whenever possible. If right-sided landmarks could not be reliably identified, all measurements for that skull were performed using left-sided landmarks. Maxillary measurements were recorded parallel to the FHP and the MSP and included distances between predefined landmarks. Mandibular measurements were recorded parallel to the MP and the MSP and included total mandibular length and segmental distances between dental and skeletal landmarks.

**Table 2:** Craniofacial measurements performed.

Measurement	Abbreviation	Definition
Maxilla		
Posterior Condyle to Sella*	PC–S	Linear distance between posterior condyle (PC) and sella (S)
Sella to Posterior Maxillary Tuberosity*	S–PTM	Linear distance between sella (S) and posterior maxillary tuberosity (PTM)
Posterior Maxillary Tuberosity to Prosthion (maxillary length)*	PTM–A	Linear distance between posterior maxillary tuberosity (PTM) and prosthion (A)
Posterior Maxillary Tuberosity to Maxillary Fourth Premolar*	PTM–MXPM4	Linear distance between posterior maxillary tuberosity (PTM) and distal alveolar point of the maxillary fourth premolar
Posterior Maxillary Tuberosity to Maxillary Canine	PTM–MXK9	Linear distance between posterior maxillary tuberosity (PTM) and distal alveolar point of the maxillary canine
Posterior Maxillary Tuberosity to Maxillary Third Incisor	PTM–MXI3	Linear distance between posterior maxillary tuberosity (PTM) and distal alveolar point of the maxillary third incisor
Maxillary Fourth Premolar to Maxillary Canine	MXPM4–MXK9	Linear distance between distal alveolar points of the maxillary fourth premolar and canine
Maxillary Canine to Prosthion	MXK9–A	Linear distance between distal alveolar point of the maxillary canine and prosthion (A)
Mandible		
Posterior Condyle to B-point (Total Mandibular Length)*	PC–B	Linear distance between posterior condyle (PC) and B-point (B), measured parallel to the mandibular plane
Posterior Condyle to Mandibular Third Molar (Ramus Length)	PC–MNM3	Linear distance between posterior condyle (PC) and distal alveolar point of the mandibular third molar, measured parallel to the mandibular plane
Mandibular Third Molar to Mandibular First Molar	MNM3–MNM1	Linear distance between distal alveolar points of the mandibular third and first molars, measured parallel to the mandibular plane
Mandibular First Molar to Mandibular Canine	MNM1–MNK9	Linear distance between distal alveolar points of the mandibular first molar and canine, measured parallel to the mandibular plane
Mandibular Canine to B-point	MNK9–B	Linear distance between distal alveolar point of the mandibular canine and B-point (B), measured parallel to the mandibular plane

* Denotes landmarks utilized in the Wylie analysis.

### 2.5 Wylie analysis and statistical grouping

Based on preliminary data analysis revealing significant sexual dimorphism, patients were stratified by sex. Of the 146 skulls, 96 (47 males, 49 females) were selected at random as a group to establish sex-specific normative datasets (normalization group). The remaining 50 skulls (24 males, 26 females) were assigned to a validation group for the Wylie analysis. Five measurements were utilized for the Wylie analysis (Figure 2) (Table 2). Four measurements: Posterior Condyle to Sella (PC–S), Posterior Condyle to Posterior Maxillary Tuberosity (PC – PTM), Posterior Maxillary Tuberosity to Prosthion (PTM –A), and Posterior Maxillary Tuberosity to maxillary fourth premolar (PTM –MXPM4) are considered maxillary measurements. The remaining measurement is the mandibular measurement. The difference for each of the five measurements between the reference standard (the mean value acquired from the normative group) and each patient in the validation group were calculated according to the methods established by Wylie (9) using Equations 1, 2:


Maxillary Difference=Standard–Patient measurement (1)

Mandibular Difference=Patient measurement–Standard (2)

An example of how the Wylie analysis is implemented is found in (Table 3). For maxillary measurements, when the patient measured distance is smaller than the reference value, a positive value is obtained; its absolute value is placed in the “Favors Class 3” column. When the patient measured distance is greater than the reference value, a negative value is obtained; its absolute value is placed in the “Favors Class 2” column.

**Table 3:** This table illustrates how the Wylie analysis was performed using the example of a female Labrador retriever from the validation group.

Dimension	Reference standard	Patient	Favors class 2 (Column A)	Favors class 3 (Column B)
Posterior Condyle – Sella (PC–S)	9.69	11.25	1.56	
Sella – Posterior Maxillary Tuberosity (S–PTM)	43.27	41.64		1.63
Posterior Maxillary Tuberosity – Prosthion (PTM–A) *(Maxillary Length)*	101.50	99.92		1.58
Posterior Maxillary Tuberosity – Maxillary Fourth Premolar (PTM–MXPM4)	16.86	16.13		0.73
Posterior Condyle – B-point (PC–B) *(Mandibular Length)*	154.52	153.24	1.28	
Totals			2.84	3.94
Wylie score			1.1	

The first column lists the reference standard values (mean) in millimeters derived from the normative group. The column labeled “Patient” contains the measurements obtained from the individual patient. The difference between the reference standard value and the patient’s values are calculated using the following formulas:

Equation 1: Maxillary Difference = Standard – Patient.

Equation 2: Mandibular Difference = Patient – Standard.

The absolute values from each equation are placed into Favors Class 2 column (Column A) if the value is negative, and Favors Class 3 column (Column B) if the value is positive. The Wylie score is then calculated by subtracted Column a total from Column B total. In this example, the result is 1.1, which favors a Class 3 malocclusion.

For the mandibular measurements, when the patient measured distance is greater than the reference value, a positive value is obtained; its absolute value is placed in the “Favors Class 3” column. When the patient measured distance is lower than the reference value, a negative value is obtained; its absolute value is placed in the “Favors Class 2” column.

All values in each column are totaled. Lastly, the Wylie score is calculated by subtracting the “Favors Class 2” column total from the “Favors Class 3” column total (Equation 3). The Wylie score is a measure of maxillary to mandibular length discrepancy.


Wylie score=Favors Class3total–Favors Class2total (3)

### 2.6 Facial angle analysis

Angular measurements were performed in the midsagittal plane. The SNA (Sella–Nasion–Prosthion/A-point) and SNB (Sella–Nasion–B-point) angles were calculated to assess anteroposterior positions of the maxilla and mandible relative to the cranial base, respectively (Figure 5). The ANB angle, which provides a relative value representing the maxillary to mandibular relationship, was calculated as the difference between SNA and SNB and recorded for each patient.

**Figure 5:**
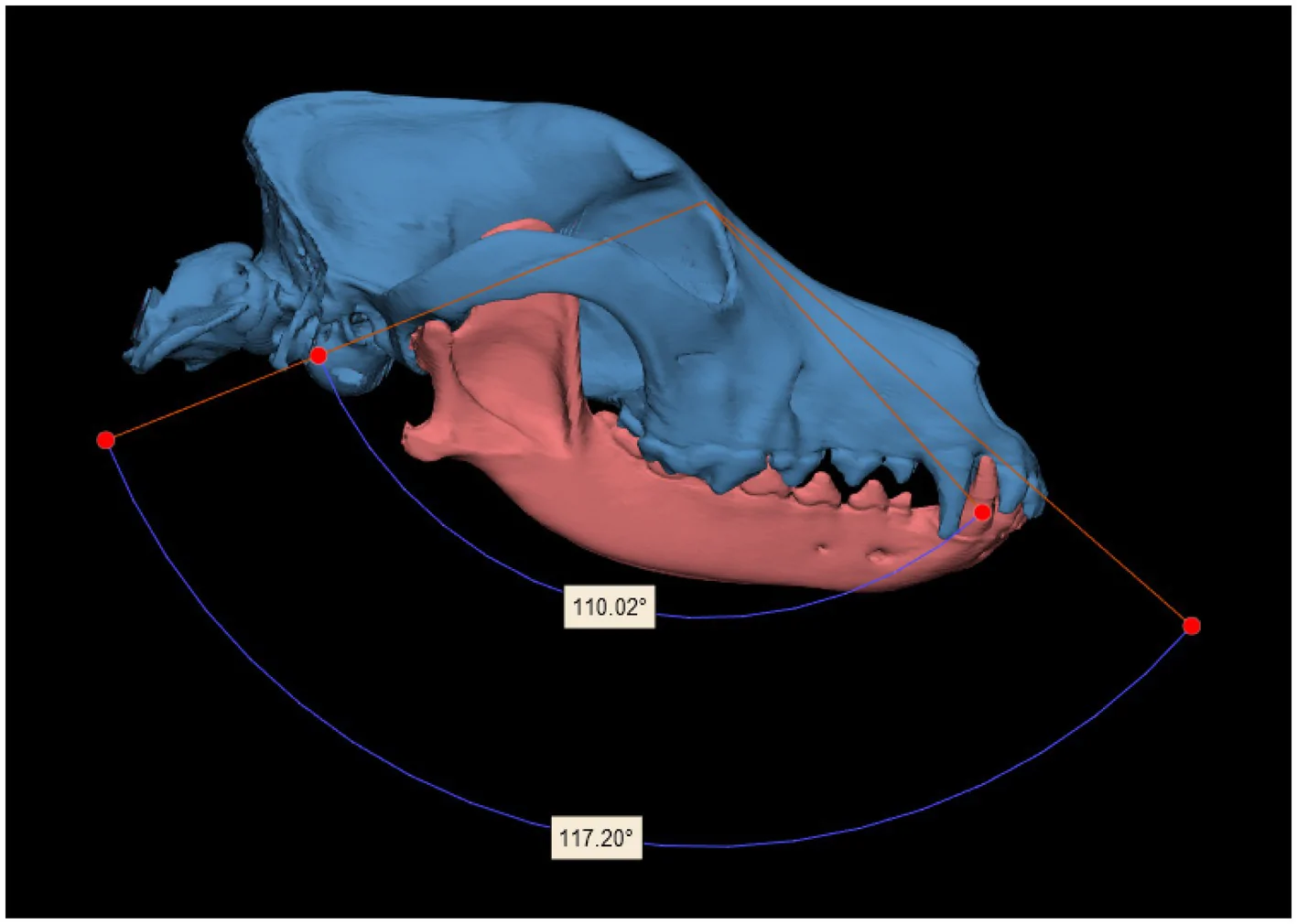
Representation of the SNA and SNB angular measurements. In this case, the SNA is 117.2° and the SNB is 110.02°.

### 2.7 Ratio-based analysis

Additional ratios were calculated to assess maxillomandibular relationships. These included the following ratios:

1. The distance from the posterior condyle to the maxillary fourth premolar over the distance from the posterior condyle to the mandibular first molar (MXPM4: MNM1)
2. The distance from the posterior condyle to the maxillary canine over the distance from the posterior condyle to the mandibular canine (MXK9: MNK9)
3. The total maxillary length over the total mandibular length (MX: MN).

Descriptive statistics were calculated for each ratio by sex.

### 2.8 Statistical analysis

Descriptive statistics were performed on the results from craniofacial measurements, Wylie scores, facial angles analysis, and ratio-based analyses. Data are summarized by mean (SD or 95% CI), median (range), N (%), or slope estimate (95% CI). Prior to performing cephalometric analyses, the mean values of each craniofacial measurement between males and females were compared, and Holm’s corrected *p*-value noted significant difference between sexes for all but three mean craniofacial measurements. This significant difference between males and females for most measurements determined the use of sex stratified standards of all measurements in the Wylie analysis. Comparisons between groups were made via T-test or Chi-square test. Linear regression analysis was used to examine the relationship between craniofacial measurements and weight while controlling for sex as a covariate. Similarly, linear regression analysis estimated the slope (95% CI) of continuous predictors and Wylie score as the outcome. All analyses were conducted using R for Statistical computing version 4.2. Significance level was set at 0.05.

## 3 Results

A total of 146 head CT studies of Labrador retrievers met the inclusion criteria. Study patients included 75 females [8 intact (5.5%), 67 spayed (45.9%)] and 71 males [12 intact (8.2%), 59 castrated (40.4%)]. The mean (± SD) body weight was 35.6 (±7.6) kg.

### 3.1 Craniofacial measurements

Descriptive statistics for all craniofacial measurements are provided in Figure 6 and Table 4. When comparing the mean (± SD) between the normalization and validation subsets, there was no significant difference between groups. With these results, we were able to use the normalization group to set a standard in which to compare the validation subset of cases.

**Figure 6:**
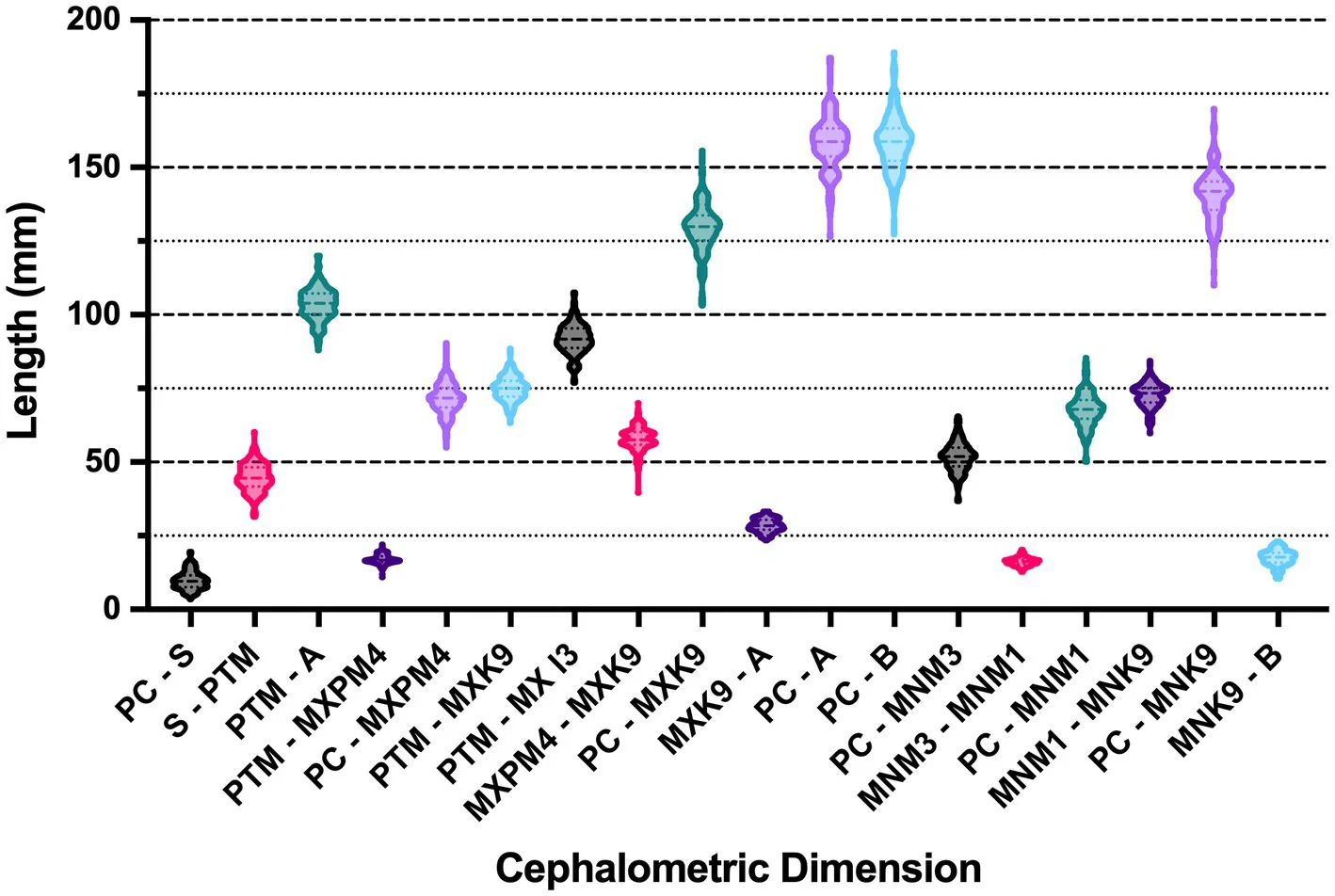
Violin plots depicting median, 1st and 3rd quartiles, min and max as well as distribution of all cephalometric dimensions in the present study.

**Table 4:** Craniofacial measurements in millimeters means and SD between normalization and validation groups.

Variable	All patients (*n* = 146) Mean (SD)	Normalization group (*n* = 96) Mean (SD)	Validation group (*n* = 50) Mean (SD)	Adj P
PC - S	9.9 (3.1)	9.8 (3.0)	10.1 (3.2)	1
S - PTM	44.8 (4.7)	44.9 (4.4)	44.5 (5.2)	1
PTM - A	103.6 (5.5)	103.9 (5.4)	103.1 (5.7)	1
PTM - MXPM4	17.0 (1.7)	17.0 (1.8)	17.1 (1.5)	1
PC - MXPM4	71.7 (5.6)	71.7 (5.3)	71.7 (6.1)	1
PTM - MXK9	75.0 (4.2)	75.1 (4.1)	74.8 (4.4)	1
PTM - MXI3	91.9 (5.2)	92.1 (5.1)	91.5 (5.3)	1
MXPM4 - MXK9	57.9 (4.0)	57.9 (4.1)	57.7 (4.1)	1
PC - MXK9	129.6 (8.3)	129.6 (8.0)	129.4 (8.8)	1
MXK9 - A	28.6 (2.3)	28.7 (2.3)	28.4 (2.3)	1
PC - A	158.3 (9.3)	158.6 (8.9)	157.7 (10.2)	1
PC - B	158.1 (9.7)	158.5 (9.1)	157.4 (10.7)	1
PC - MNM3	51.8 (4.9)	52.0 (4.6)	51.5 (5.6)	1
M3 - MNM1	16.1 (1.4)	16.0 (1.2)	16.2 (1.6)	1
PC - MNM1	67.9 (5.8)	68.0 (5.3)	67.7 (6.6)	1
M1 - MNK9	72.8 (4.1)	73.0 (3.8)	72.2 (4.5)	1
PC - MNK9	140.6 (8.8)	141.0 (8.1)	139.9 (10.1)	1
K9 - B	17.5 (2.7)	17.6 (2.8)	17.4 (2.4)	1

*p*-value reflects the difference in measurements between the groups.

#### 3.1.1 Sexual dimorphism in craniofacial measurements

Craniofacial measurements differentiated by sex are provided in Table 5. Males had consistently larger linear dimensions than females across both maxillary and mandibular measurements. Specifically, 14 of 18 measurements showed significant sex differences, with the exceptions of PC–S, PTM–MXPM4, MNM3–MNM1, and MNK9–B. Given this sexual dimorphism, sex-specific reference standards were established for all subsequent analyses.

**Table 5:** Craniofacial measurements in millimeters in mean and SD comparison between male and female patients.

Variable	Female (*n* = 75) Mean (SD)	Male (*n* = 71) Mean (SD)	Adj P
PC - S	9.8 (3.2)	10.0 (3.0)	1
S - PTM	43.3 (4.3)	46.3 (4.7)	0.002
PTM - A	101.4 (4.8)	106.0 (5.2)	< 0.001
PTM - MXPM4	16.9 (1.7)	17.2 (1.7)	1
PC - MXPM4	70.1 (5.2)	73.4 (5.5)	0.003
PTM - MXK9	73.5 (3.8)	76.6 (4.1)	< 0.001
PTM - MXI3	89.8 (4.5)	94.0 (5.0)	< 0.001
MXPM4 - MXK9	56.4 (3.8)	59.4 (3.7)	< 0.001
PC - MXK9	126.5 (7.4)	132.8 (7.9)	< 0.001
MXK9 - A	27.9 (2.2)	29.3 (2.1)	0.002
PC - A	154.5 (8.0)	162.2 (9.0)	< 0.001
PC - B	154.5 (8.4)	162.0 (9.5)	< 0.001
PC - MNM3	50.2 (4.6)	53.5 (4.8)	0.001
MNM3 - MNM1	15.9 (1.3)	16.2 (1.4)	1
PC - MNM1	66.2 (5.3)	69.6 (5.8)	0.003
MNM1 - MNK9	71.2 (3.8)	74.4 (3.8)	< 0.001
PC - MNK9	137.4 (7.9)	144.0 (8.5)	< 0.001
MNK9 - B	17.1 (2.5)	17.9 (2.9)	0.719

#### 3.1.2 Allometry in craniofacial measurements

After controlling for sex, 14 of 18 craniofacial measurements showed significant allometry. Measurements not significantly associated with weight were PC–S, PTM–MXPM4, MNM3–MNM1, and MNK9–B (Table 6).

**Table 6:** Analysis of correlation between individual craniofacial measurements and patient weight.

Variable	Slope (95% CI)*	Adj. P	Partial R-squared
PC - S	−0.047 (−0.115, 0.021)	1	0.017
S - PTM	0.334 (0.232, 0.436)	< 0.001	0.24
PTM - A	0.369 (0.254, 0.484)	< 0.001	0.205
PTM – MXPM4	0.041 (−0.001, 0.084)	0.454	0.032
PC - MXPM4	0.328 (0.201, 0.455)	< 0.001	0.164
PTM – MXK9	0.227 (0.129, 0.324)	< 0.001	0.133
PTM – MXI3	0.292 (0.177, 0.407)	< 0.001	0.148
MXPM4 - MXK9	0.208 (0.111, 0.305)	< 0.001	0.119
PC - MXK9	0.536 (0.352, 0.720)	< 0.001	0.186
MXK9 - A	0.136 (0.084, 0.189)	< 0.001	0.167
PC - A	0.656 (0.460, 0.851)	< 0.001	0.219
PC - B	0.611 (0.397, 0.826)	< 0.001	0.182
PC - MNM3	0.338 (0.228, 0.448)	< 0.001	0.212
MNM3 - MNM1	0.045 (0.010, 0.080)	0.116	0.054
PC - MNM1	0.383 (0.253, 0.513)	< 0.001	0.205
MNM1 - MNK9	0.216 (0.123, 0.308)	< 0.001	0.134
PC - MNK9	0.599 (0.402, 0.795)	< 0.001	0.204
MNK9 - B	0.025 (−0.044, 0.094)	1	0.005

*Denotes 95% CI reported as estimated change in corresponding variable after controlling for Sex.

### 3.2 Wylie analysis

The mean (± SD) Wylie score for males and females, respectively were males: −0.23 ± 2.66 mm and females: −0.33 ± 3.34 mm. All individuals had Wylie scores that fell into “Favors Class 2” (*N* = 25) or “Favors Class 3” (*N* = 25) categories. There was no significant difference in the Wylie score between sexes (*p* = 0.902) (Supplementary Tables S1, S2) (Figure 7). Additionally, there was no significant association between the Wylie score and body weight after controlling for sex (slope = −0.045, *p* = 0.497; partial r^2^ = 0.012) (Figure 7).

**Figure 7:**
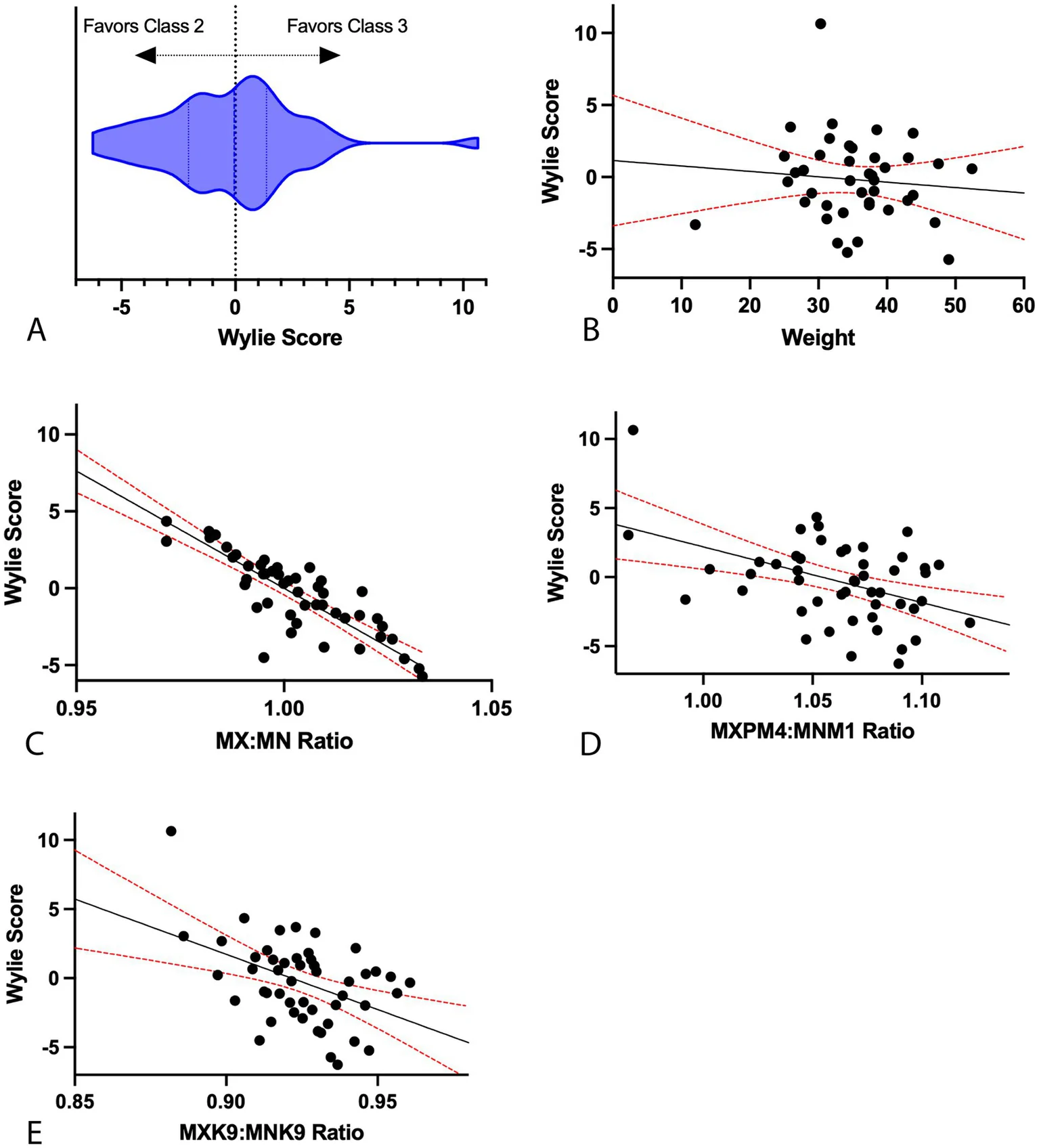
**(A)** Violin plot depicting all Wylie scores in the validation group. The vertical dashed line represents the hypothesized Wylie score (0) if maxillary length is exactly equal to mandibular length. All scores below 0 would tend to favor a Class 2 occlusion and all scores above 0 would tend to favor a Class 3 occlusion. In the plot, this line is very close to the median score. The smaller dashed lines within the violin plot represent the 1^st^ and 3^rd^ quartiles. Regression plots with red dashed lines representing the 95% CI depict the between Wylie score and weight in kg **(B)**, MX: MN ratio **(C)**, MXPM4: MNM1 ratio **(D)**, and MXK9: MNK9 ratio **(E)** are also presented.

### 3.3 Facial angle analysis

The mean (± SD) SNA, SNB, and ANB angles, respectively, were 115.1° ± 4.3, 107.2° ± 4.2, and 7.8° ± 1.5 (Figure 8). There was no significant difference in any facial angle measurement between the normalization and validation groups or between male and female patients. In addition, no significant associations were found between body weight and any facial angle measurement after controlling for sex.

**Figure 8:**
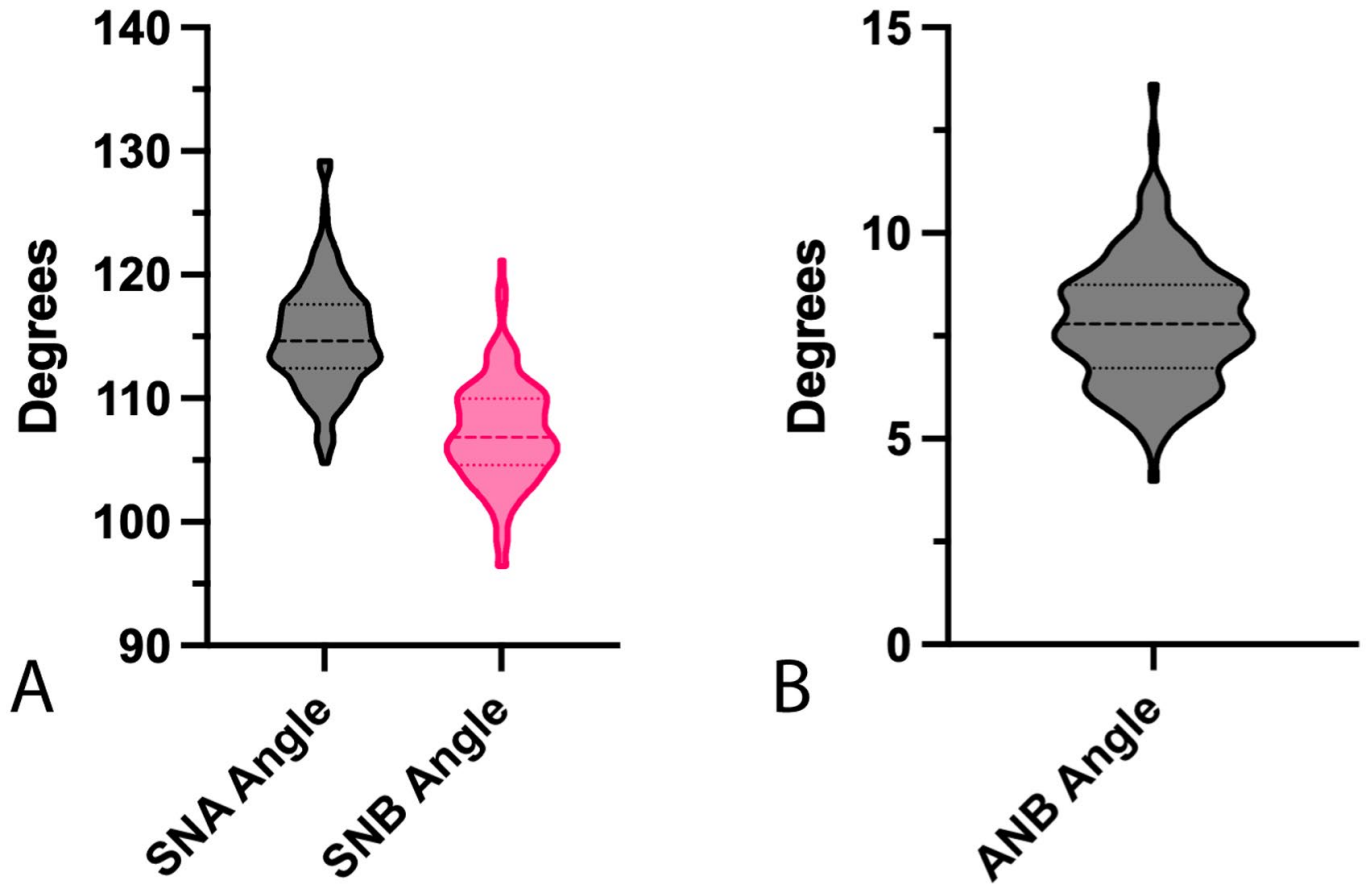
Violin plots depicting the median, 1st and 3rd quartiles and distribution of the SNA and SNB angles **(A)** as well as the ANB angle **(B)**.

### 3.4 Ratio-based analysis

The mean MXPM4: MNM1 ratio was 1.1, the MXK9: MNK9 ratio was 0.9, and the MX: MN ratio was 1.0 across all individuals (Figure 9). No significant differences were found between the normalization and validation groups for any ratio. Sexual dimorphism was not observed in any of these ratio-based measurements. Allometry was observed in the MXPM4: MNM1 ratio (slope = −0.001; adjusted *p* = 0.036; partial r^2^ = 0.074) (Figure 9).

**Figure 9:**
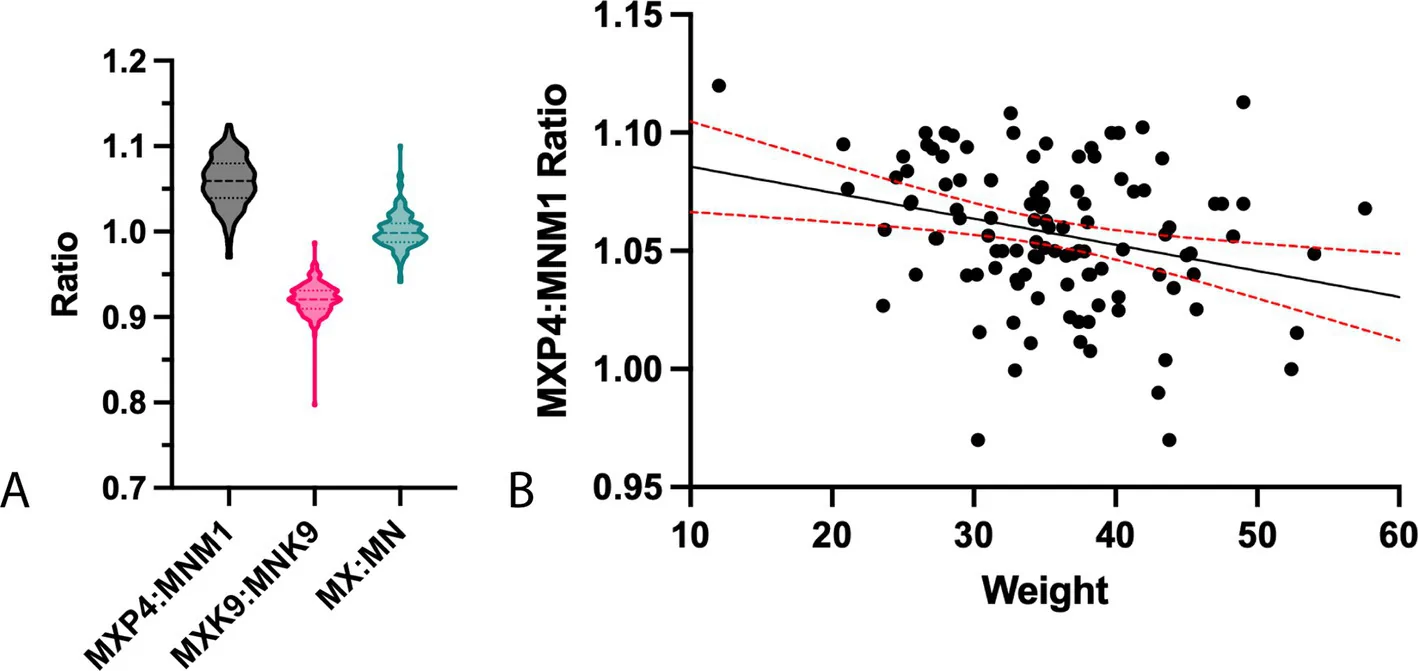
**(A)** Violin plots depicting the median, 1st and 3rd quartiles and distribution of the MXPM4: MNM1, MXK9: MNK9 and MX: MN ratios. **(B)** Scatter plot depicting the correlation between MXPM4: MNM1 ratio and patient body weight. The dashed red lines in the scatter plots represent the 95% CI.

### 3.5 Comparison of Wylie, angle, and ratio analyses

The Wylie score was strongly and significantly correlated with all three ratio-based measurements. The strongest correlation was observed with the MX: MN ratio (partial r = 0.866; *p* < 0.001), followed by the MXPM4: MNM1 ratio (partial r = 0.465; *p* = 0.001) and MXK9: MNK9 ratio (partial r = 0.433; *p* = 0.002) (Figure 7). There was no significant correlation between the Wylie score and the ANB angle (partial r = 0.239; *p* = 0.098).

## 4 Discussion

Currently, veterinary medicine lacks a quantitative system to analyze and document occlusion in dogs. The goal of this study was to validate cephalometric methods as the first step toward developing an objective approach to assess and characterize occlusion in individual patients. We adapted principles of human cephalometric analysis to three-dimensional CT-based models in mesaticephalic dogs and established quantitative reference standards for maxillomandibular relationships in Labrador Retrievers with clinically normal occlusion. Collectively, these findings support the concept that quantitative cephalometric analysis can address major limitations of the currently subjective and descriptive approaches used in veterinary occlusal assessment.

By randomizing patients into separate groups, we were able to internally validate our methods. There was no significant difference in craniofacial measurements between our normalization group and validation groups, indicating that the mean values acquired from the normalization group can serve as a reference standard for comparison. In addition, the lack of significant difference between the normalization and the validation groups in the ratio-based and angular analyses suggests that evaluation across the samples was consistent.

A principal finding of this study was significant sexual dimorphism, with males having larger linear dimensions than females, even after correcting for weight, emphasizing the importance of creating sex-stratified reference standards. Weight also significantly affected most craniofacial measurements, consistent with a significant allometric relationship. This is consistent with previous reports of carnivore facial length showing positive scaling with body weight (23). Notably, most ratio-based measurements remained stable across increased weight, suggesting that functional occlusal relationships are conserved across body sizes. This finding likely reflects strong evolutionary and biomechanical pressures maintaining efficient occlusal function.

At this time, there is no formal resource or measurement to assess the length of the maxilla to the mandible in the veterinary field. Amongst all the veterinary literature that aims to describe malocclusion, the relationship of the dental arch is prioritized, and little is described regarding the maxillomandibular skeletal relationship. We hypothesized that the mean Wylie score in dogs with normal occlusion would approximate zero, which assumes that the total maxillary length would be equivalent to the total mandibular length. No patient in the present study had a Wylie score of exactly zero. The mean Wylie score for males and females was <0.5 mm. Rounding our mean score to the nearest whole number yields a mean Wylie score of 0 (SD = 3), supporting our hypothesis that patients with clinically normal occlusion would, on average, have zero maxillary-to-mandibular discrepancy. The Wylie score standard deviation was three millimeters, which we suspect is small enough to be undetectable on routine examination. All individual patient scores within the validation group favored either a Class 2 (mandibular disocclusion) or Class 3 (mandibular mesioclusion) relationship. In humans, the Wylie analysis is measured in whole numbers, whereas the 3-Matic software used in this study measured to the hundredth of a millimeter, allowing more precise measurement (9). Overall, these findings likely represent the phenomenon of dental compensation in which dental eruption pathways can accommodate minor skeletal imbalances. Dentofacial compensation is well-described in human orthodontics and may mask underlying skeletal imbalances to maintain functional occlusion and facial harmony (13). We also identified several outlier patients with large Wylie scores but clinically normal occlusion, supporting the possibility that dentofacial compensation is present in our patient population.

The second cephalometric analysis to assess maxillary to mandibular relationships was the facial angles analysis, which demonstrated that canine cephalometric relationships differ substantially from established human standards. In humans, a normal calculated ANB angle is approximately 2°, with a standard deviation of ± 2°. Patients with ANB angles larger than 4° are considered to have a Class 2 malocclusion, while patients with ANB angles less than 2° are considered to have a Class 3 malocclusion (8). The mean ANB angle calculated in this study was 7.8° ± 1.5°, indicating that human angular reference standards cannot be directly applied to dogs. This discrepancy is likely attributable to the distinct morphology of the canine cranial base and midface. In other words, the relevant anatomic landmarks occupy unique positions in dogs, and a larger ANB angle is likely normal in mesaticephalic dogs. We would anticipate dogs with a Class 2 malocclusion will have an ANB angle larger than approximately 9.3° (7.8° + 1.5°); dogs with ANB angles less than approximately 6.3° would likely be characterized by a Class 3 malocclusion. Larger comparison studies of these populations would be needed to verify these proposed measurements.

Additionally, we created ratio-based assessments using previously described craniofacial measurements to gain insight into relative regional contributions to overall maxillary and mandibular lengths. In a patient with normal occlusion, we would expect that the MXPM4: MNM1 ratio would be slightly greater than one, as the mandibular first molar should be located distal to the maxillary fourth premolar tooth in the horizontal plane (1, 2, 24). A previous study assessing this relationship in mesaticephalic and brachycephalic dogs reported a mean maxillary-to-mandibular carnassial length ratio of 1.2 in mesaticephalic dogs however, that method measured from the external ear (Porion) to the cusp of the carnassial teeth without the use of a horizontal reference plane (25). In contrast, our method uses the FHP to approximate natural head position, and measures from the condyle to the alveolar process of the respective teeth, removing the variability introduced by tooth angulation. While these measurements are similar, they cannot be directly compared due to these differences.

The MXPM4: MNM1 measurement is analogous to the Angle classification of occlusion in humans, that defines malocclusion classes by the relationship of the mesiobuccal cusp of the maxillary first molar tooth occluding with the buccal groove mandibular first molar teeth (8). Our goal was to provide a consistent, quantitative measurement of this relationship, which offers insight into both the dental occlusion and the skeletal occlusion at this location. Across both the normalization and validation groups, the ratio is slightly greater than one, confirming our hypothesis that this relationship is consistent with normal occlusion. In multiple anatomic studies assessing carnassial relationships, it is noted that this relationship is highly conserved across carnivore species (26, 27). Although maxillomandibular relationships and occlusion were not assessed in those studies, the consistency of the carnassial relationship between these species reinforces the theory of dentofacial compensation and achieving facial harmony, in this case, for maintaining functionality. This is the only ratio noted to scale allometrically; this is likely a result of a highly conserved relationship of the carnassial teeth. The MXPM4: MNM1 ratio creates a quantitative reference standard for future use.

We hypothesized that the MXK9: MNK9 ratio should be slightly less than one, as the mandibular canine should sit mesial to the maxillary canine in the interdental space between maxillary canine and the third incisor (1). This was confirmed by our findings in both the normalization and validation groups, and a mean ratio of 0.9 may be considered a reference value in Labrador Retrievers for future studies.

Similarly, we postulated that the MX: MN ratio should be equal to one, as the maxilla should align with the mandible. Our results are consistent with our hypothesis. As noted above, no formal studies have assessed the exact length or relationship between the maxilla and mandible; this study therefore establishes a reference standard for normal occlusion in mesaticephalic patients.

The overarching goal with the ratio-based measurements was to create a clinically intuitive framework and metric for assessment of occlusion. By evaluating three distinct ratios, each maxillomandibular relationship can be assessed individually, which may help localize the specific portion of the maxilla or mandible contributing to a discrepancy. This assessment should be validated in future studies of Class 2 and Class 3 patients.

All ratio-based measurements correlated strongly with the Wylie score, further validating the internal consistency of our measurements and analyses. The strong relationship between the Wylie score and the MX: MN ratio is likely due to overlap in the craniofacial measurements used in each analysis; these approaches capture the same information regarding skeletal occlusion, with the Wylie score providing greater regional anatomic detail.

In contrast, facial angles analysis did not correlate with Wylie scores, which may reflect the influence of vertical dimensions that are not incorporated into the Wylie analysis (28). While this is a major criticism of the sole use of the Wylie analysis in humans, we believe the methodology borrowed from the Wylie analysis is uniquely suited for canine occlusal assessment. While vertical dysplasia can contribute significantly to human malocclusion assessment and diagnosis, the head position and shape of dogs are oriented primarily in the horizontal plane (8, 28). A historically common method to characterize canine skull morphology relies on horizontal measurements to generate a cephalic index, placing patients into one of three craniofacial morphologies: dolichocephalic, mesaticephalic, and brachycephalic (29, 30). Discrepancies in the vertical dimension may not be as significant in mesaticephalic or dolichocephalic patients but could be an important consideration in brachycephalic patients that are affected by sinus aplasia and other skull abnormalities (31).

In human cephalometry, the FHP was developed to measure natural head position with the cranial base parallel to the floor. The original description references forward gaze (eyes directed toward the horizon) as the reference orientation (19, 32). Studies of natural head position in mammals, including dogs, demonstrate that the lateral semicircular canals should lie horizontal, an orientation represented by the FHP (20). Specific anatomic landmarks – Porion and Orbitale – can be difficult to reproduce, this method remains consistent and is used even in three-dimensional cephalometric evaluation (18, 33).

Landmark assignment in our study is similar to that used in human lateral cephalograms, and the detailed descriptions of anatomic landmarks are easily transferred from human cephalometry. By adopting an established methodology for identification of canine craniofacial anatomy, we provide a uniform system of measurement and assessment that reduces the subjectivity of current methods of occlusal evaluation. Three-dimensional imaging, especially cone beam CT (CBCT), is increasingly utilized in both human and veterinary dental practice (17, 34, 35). Metanalyses assessing traditional 2D cephalometry and 3D cephalometry show that the techniques are comparable (14, 36). By using 3D imaging, such as conventional or cone beam CT, we decrease errors with positioning in our canine patients. In humans, dedicated positioning devices are used to prevent rotation and help standardize cephalograms; the development of analogous devices for veterinary patients would not be feasible given the marked variation in skull morphology. Therefore, validating methods of cephalometry for use with 3D images in our canine patients is necessary.

In this study, we aim to redefine the current methods of occlusion assessment in dogs, which remain primarily subjective and qualitative in nature. Although the AVDC definitions of normal occlusion in dogs have recently been updated to include a description of the mesiodistal relationship of the carnassial teeth, the absence of quantitative measurement leaves room for error. Additionally, the current system does not account for differences in dental angulation, which can affect occlusion diagnosis due to the phenomenon of dentofacial compensation. By providing an objective framework for measuring skeletal and dental relationships, our approach can be used to identify and classify malocclusions in dogs, with the potential to improve diagnostic standardization, interobserver consistency, and treatment planning.

Several limitations of this study should be acknowledged. The retrospective design relied on previous medical records for oral examination findings and introduced variability in CT scanner and technique, which may have produced minor differences in landmark identification precision owing to differences in resolution. However, the lack of significant difference in craniofacial distances between groups suggests that image resolution did not severely impact our ability to place anatomical landmarks accurately and consistently. Currently, the cephalometric methods were only performed in a single breed, the Labrador Retriever, as a representative of mesaticephalic skulls. Future studies to assess these cephalometric analyses would be warranted using other craniofacial morphologies (dolichocephalic and brachycephalic). Additionally, future studies should include cephalometric analyses in patients with known malocclusions in mesaticephalic patients and assessing diagnostic sensitivity and specificity relative to the reference values established in this study.

The methods used in the present study were manually intensive and performed in a specialized software with somewhat limited access to those in clinical practice. However, the results of the present study could serve as the basis for incorporation into veterinary clinical imaging software. These systems already exist in the human orthodontics realm in which anatomical landmark placement, measurement and analysis is an automated feature often using AI/machine learning tools.

In conclusion, this study establishes the first quantitative framework for objective assessment of occlusion in dogs using CT-based cephalometric analysis. By adapting validated human cephalometric principles to canine craniofacial anatomy, we demonstrate that skeletal and dental relationships can be measured and expressed as clinically meaningful reference values. The Wylie analysis, facial angle analysis, and ratio-based measurements each provide complementary information regarding maxillomandibular relationships and together offer a more comprehensive assessment than existing descriptive classification systems. These methods represent an important step toward standardized diagnosis of canine malocclusion and provide a foundation for future investigations in diverse skull conformations, objective classification of malocclusion severity, orthodontic treatment planning, and ultimately veterinary orthognathic surgery. Future studies evaluating these methods across additional skull morphologies and naturally occurring malocclusions will further define their diagnostic utility and establish a foundation for veterinary orthodontic and orthognathic surgical planning.

## Data Availability

The raw data supporting the conclusions of this article will be made available by the authors, without undue reservation.
