# Supplementary material for: Adaptation and validation of the Wylie cephalometric analysis for three-dimensional assessment of canine occlusion in Labrador retrievers
Source: Front Vet Sci. 2026 Sep 16;13:1955587. doi: 10.3389/fvets.2026.1955587 (PMC13624093; doi:10.3389/fvets.2026.1955587)
Supplement: Supplementary file 1 [file Table_1.DOCX]

**Table S1:** Wylie analysis scores for male and female patients.

| **Sex** | **PC - S** | **S - PTM** | **PTM - A** | **PTM – MXPM4** | **PC - B** | **Wylie score** |
| --- | --- | --- | --- | --- | --- | --- |
| M | 14.75 | 38.54 | 94.51 | 16.61 | 147.99 | 0.89 |
| M | 6.90 | 60.33 | 120.08 | 18.44 | 189.02 | 0.58 |
| M | 16.03 | 52.54 | 110.82 | 19.75 | 180.57 | -1.26 |
| M | 15.67 | 53.65 | 116.41 | 21.04 | 183.18 | -6.26 |
| M | 7.50 | 45.50 | 110.23 | 18.63 | 164.78 | 0.23 |
| M | 5.33 | 47.62 | 108.15 | 16.80 | 157.43 | -3.16 |
| M | 13.34 | 39.98 | 104.40 | 16.84 | 159.93 | 2.68 |
| M | 10.35 | 50.47 | 106.23 | 17.09 | 169.01 | 2.18 |
| M | 9.03 | 45.49 | 101.96 | 19.13 | 156.01 | -2.29 |
| M | 4.97 | 47.98 | 107.57 | 16.23 | 160.78 | 1.34 |
| M | 9.85 | 47.60 | 111.75 | 19.19 | 174.13 | 3.05 |
| M | 10.48 | 40.25 | 102.45 | 17.05 | 152.67 | -0.25 |
| M | 8.35 | 44.12 | 107.03 | 16.10 | 155.81 | -2.48 |
| M | 9.47 | 43.37 | 102.63 | 16.22 | 156.21 | 1.83 |
| M | 14.29 | 47.32 | 104.16 | 16.67 | 168.82 | 3.69 |
| M | 8.43 | 50.61 | 103.14 | 17.81 | 156.95 | -5.73 |
| M | 9.13 | 49.75 | 105.85 | 17.21 | 165.55 | 0.92 |
| M | 7.43 | 49.15 | 107.55 | 16.14 | 161.20 | -1.76 |
| M | 9.21 | 43.62 | 103.64 | 16.22 | 156.03 | 0.65 |
| M | 9.47 | 37.84 | 98.31 | 16.29 | 148.07 | 3.47 |
| M | 7.84 | 46.48 | 100.40 | 14.67 | 151.86 | -0.22 |
| M | 13.83 | 37.29 | 94.98 | 16.26 | 144.73 | -0.32 |
| M | 12.51 | 44.13 | 109.34 | 15.86 | 164.63 | 0.1 |
| M | 7.16 | 32.98 | 94.41 | 17.20 | 131.13 | -3.31 |
| F | 3.42 | 48 | 102.11 | 14.95 | 152.16 | 0.48 |
| F | 9.78 | 49.61 | 100.3 | 16.43 | 157.38 | -1.94 |
| F | 18.81 | 35.95 | 98.01 | 19.18 | 151.31 | -3.84 |
| F | 14.95 | 38.14 | 94.35 | 16.54 | 146.09 | -1.09 |
| F | 16.51 | 38.64 | 104.26 | 17.11 | 158.61 | -1.11 |
| F | 7.09 | 49.44 | 108.17 | 18.43 | 165.36 | -0.97 |
| F | 8.2 | 51.58 | 100.31 | 16.59 | 160.82 | 0.94 |
| F | 9.16 | 44.97 | 100.47 | 16.95 | 159.1 | 4.35 |
| F | 9.05 | 49.9 | 110 | 16.34 | 166.87 | -1.62 |
| F | 7.25 | 41.94 | 99.35 | 16.21 | 151.24 | 3.29 |
| F | 10.58 | 44.09 | 104.3 | 17.9 | 156.12 | -3.95 |
| F | 9.25 | 46.44 | 103.06 | 16.77 | 160.73 | 2.01 |
| F | 10.24 | 39.24 | 98.59 | 14.55 | 147.16 | 1.34 |
| F | 10 | 38.72 | 96.29 | 16.17 | 144.86 | 0.48 |
| F | 12.39 | 45.51 | 99.42 | 18.28 | 157.07 | -1.73 |
| F | 11.25 | 41.64 | 99.92 | 16.13 | 153.24 | 1.1 |
| F | 9.86 | 43.05 | 101.38 | 15.79 | 163.92 | 10.64 |
| F | 5.96 | 39.51 | 94.5 | 15.7 | 136.89 | -1.98 |
| F | 10.85 | 41 | 99.85 | 16.13 | 152.55 | 1.52 |
| F | 7.67 | 39.43 | 91.08 | 16.56 | 139.38 | 1.44 |
| F | 13.5 | 42.48 | 102.75 | 16.92 | 154.26 | -4.59 |
| F | 9.27 | 43.73 | 107.46 | 22.09 | 161.24 | -4.51 |
| F | 7.54 | 45.42 | 101.44 | 16.5 | 154.41 | 0.31 |
| F | 11.02 | 47.73 | 101.13 | 16.63 | 158.64 | -1.07 |
| F | 10.97 | 39.44 | 105.13 | 17.13 | 150.64 | -5.23 |
| F | 6.91 | 44.32 | 104.41 | 19.43 | 155.37 | -2.9 |

**Table S2:**  Wylie analysis score as a predictor of skeletal malocclusion in the validation cohort according to patient sex.

| **Male** | | **Female** | |
| --- | --- | --- | --- |
| **Wylie score** | **Favors Class 2 or Favors Class 3** | **Wylie score** | **Favors Class 2 or Favors Class 3** |
| 0.89 | 3 | 0.48 | 3 |
| 0.58 | 3 | -1.94 | 2 |
| -1.26 | 2 | -3.84 | 2 |
| -6.26 | 2 | -1.09 | 2 |
| 0.23 | 3 | -1.11 | 2 |
| -3.16 | 2 | -0.97 | 2 |
| 2.68 | 3 | 0.94 | 3 |
| 2.18 | 3 | 4.35 | 3 |
| -2.29 | 2 | -1.62 | 2 |
| 1.34 | 3 | 3.29 | 3 |
| 3.05 | 3 | -3.95 | 2 |
| -0.25 | 2 | 2.01 | 3 |
| -2.48 | 2 | 1.34 | 3 |
| 1.83 | 3 | 0.48 | 3 |
| 3.69 | 3 | -1.73 | 2 |
| -5.73 | 2 | 1.1 | 3 |
| 0.92 | 3 | 10.64 | 3 |
| -1.76 | 2 | -1.98 | 2 |
| 0.65 | 3 | 1.52 | 3 |
| 3.47 | 3 | 1.44 | 3 |
| -0.22 | 2 | -4.59 | 2 |
| -0.32 | 2 | -4.51 | 2 |
| 0.1 | 3 | 0.31 | 3 |
| -3.31 | 2 | -1.07 | 2 |
|  |  | -5.23 | 2 |
|  |  | -2.9 | 2 |
